# Effects of a commercially formulated glyphosate solutions at recommended concentrations on honeybee (*Apis mellifera* L.) behaviours

**DOI:** 10.1038/s41598-020-80445-4

**Published:** 2021-01-22

**Authors:** Qi-Hua Luo, Jing Gao, Yi Guo, Chang Liu, Yu-Zhen Ma, Zhi-Yong Zhou, Ping-Li Dai, Chun-Sheng Hou, Yan-Yan Wu, Qing-Yun Diao

**Affiliations:** 1 Key Laboratory of Pollinating Insect Biology, Ministry of Agriculture, Institute of Apicultural Research, Chinese Academy of Agricultural Sciences, Beijing, 100093 China; 2 Bureau of Landscape and Forestry, Mi Yun District, Beijing, 101500 China

**Keywords:** Animal behaviour, Behavioural ecology, Drug safety

## Abstract

Glyphosate, the active ingredient of the most widely used commercial herbicide formulation, is extensively used and produced in China. Previous studies have reported sublethal effects of glyphosate on honeybees. However, the effects of commercially formulated glyphosate (CFG) at the recommended concentration (RC) on the chronic toxicity of honeybees, especially on their behaviours, remain unknown. In this study, a series of behavioural experiments were conducted to investigate the effects of CFG on honeybees. The results showed that there was a significant decline in water responsiveness at 1/2 × , 1 × and 2 × the RC after 3 h of exposure to CFG for 11 days. The CFG significantly reduced sucrose responsiveness at 1/2 × and 1 × the RC. In addition, CFG significantly affected olfactory learning ability at 1/2 × , 1 × , and 2 × the RC and negatively affected memory ability at 1/2 × and 1 × the RC. The climbing ability of honeybees also significantly decreased at 1/2 × , 1 × and 2 × the RC. Our findings indicated that, after they were chronically exposed to CFG at the RC, honeybees exhibited behavioural changes. These results provide a theoretical basis for regulating field applications of CFG, which is necessary for establishing an early warning and notification system and for protecting honeybees.

## Introduction

Honeybees (*Apis mellifera* L.) are one of the most important economic insects for crop pollination. Honeybee pollination is important for crops, orchards, endangered plant survival, urban horticulture and ecological restoration, and honeybees account for more than 80% of the total number of pollinating insects in nature^1^. However, due to excessive applications of a large number of pesticides, honeybee poisoning incidents have occurred frequently in recent years^2^. Specifically, the population of honeybees in many countries throughout North America, Europe and Asia has decreased significantly because of the excessive use of pesticides^3,4^. The reduction in the number of honeybees may cause enormous economic losses in the beekeeping industry, and yields and crop quality may diminish because of insufficient pollination^5–8^. Compared with that of other insect species, the genome of honeybees lacks genes that encode detoxification enzymes, so honeybees are highly susceptible to pesticides^9^. Therefore, honeybees are the standard test organism for assessing the potential effects^10^ of pesticides on terrestrial invertebrates, and there is a set of standard methods to study this species^11–15^. In order to use pesticides safely, it is important to study the toxicity of commercial pesticide formulations to honeybees.

Glyphosate (GLY) is one of the most widely used herbicides worldwide. The glyphosate active ingredient and preparations manufactured by Chinese companies account for more than 60% of the global market^16^. Glyphosate isopropylamine salt has been at the top of sales annually. China is a major producer and consumer of glyphosate-based formulations, especially Roundup brand herbicides. Previous studies have mostly used pure glyphosate (> 99%) in experiments to test the effect on honeybees^17–20^; however, honeybees that are in direct contact with glyphosate in the field are commercially formulated glyphosate (CFG) at the recommended concentration (RC).

A reliable herbicide spraying early warning system has not yet been established in the rural areas of China (106.7 million ha of grain production area, 618.66 million rural permanent residents)^21^, which means that beekeepers in those areas are usually not informed before herbicides are sprayed. Therefore, there are frequent poisoning incidents of honeybees caused by mistaking the source of honey and pollen to which peasant farmers have just applied or are applying glyphosate-based formulations. Farmland in the rural areas of China is scattered due to differential household distribution, and the spraying of herbicides is not regular. During the spray period of commercially formulated glyphosate, alternate spraying of commercially formulated glyphosate in several plots around apiaries lasting for more than 10 days is common^22,23^. In China, honeybees may forage this herbicide at recommended concentration for a long time, but there are few studies on the adverse effects of honeybees under such conditions.

Several studies demonstrated the influence of commercially formulated glyphosate to locomotion behaviors. Roundup residues significantly reduced locomotion of beetles^24^. The escape swimming speed of damselfly larvae exposed to Roundup significantly decreased^25^. And Roundup also inhibited locomotion on the soil invertebrates, *Caenorhabditis elegans*^26^. Commercially formulated glyphosate (e.g. Roundup) also affected nerve system. Relating olfactory neurotoxicity to altered olfactory-mediated behaviors was found in rainbow trout exposed to three currently-used pesticides (including Roundup)^27,28^. In rat adult offspring, the glyphosate-based herbicide could also induce neurotoxicity^29^. For the model insect honeybees, the climbing assay could detect their locomotion^10,17,30^. The use of proboscis extension response to water and sucrose responsiveness could detect the ability of honeybees to forage water and food^17,30,31^. Besides, the effects on learning and memory in the nervous system was also important for foraging efficiency and survival of honeybees^17,30,32^. However, there were few studies on the effects of the commercially formulated glyphosate at recommended concentrations on the above-mentioned behaviors of honeybees.

On the basis of the above considerations, we used Roundup (41%), the most widely used herbicide formulation, in laboratory to study the effects of the recommended spray concentration on the water responsiveness, sucrose responsiveness, olfactory learning, memory ability and climbing ability of the honeybees after the spray period (more than 10 days). It is with hope that the results of this study can be used to evaluate the effects of commercially formulated glyphosate at the recommended concentration on honeybee behaviour during the intensive spray period and can further provide a scientific basis for the establishment of an early warning system for beekeepers in actual and better protection of honeybees.

## Results

### LD_50_

The honeybees were treated with Roundup at different concentrations, and the insect mortality was recorded within 48 h. The mortality were respectively 3.67 ± 1.13%, 7.77 ± 3.04%, 35.25 ± 7.94%, and 75.00 ± 2.85% at the concentrations of 0 g a.i./L, 0.72 g a.i./L, 3.6 g a.i./L, and 7.2 g a.i./L. The ingestion LD_50_ was 309 µg/bee (y = 5.454x − 1.684).

### Roundup decreased water responsiveness

**Figure 1:**
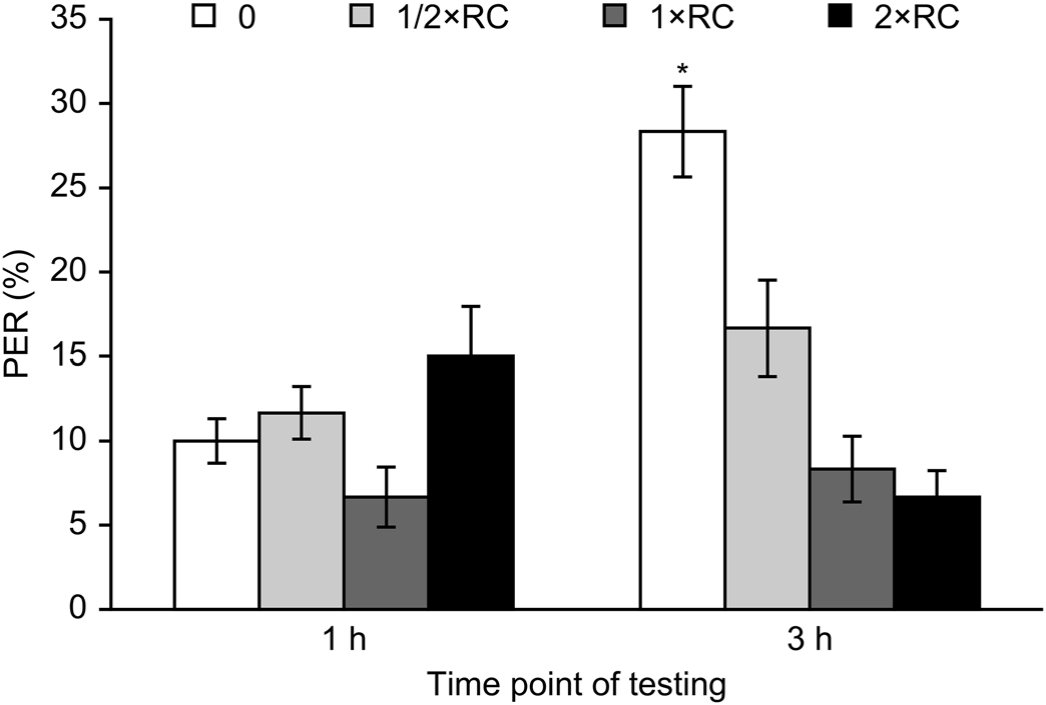
Water responsiveness of honeybees treated with Roundup. The values represent the means ± SEMs. df = 11. Significant diferences from the control are indicated by * (p < 0.05).

No significant effect of Roundup on water responsiveness was detected at 1 h after treatment (p = 0.49). Compared with the control solution, Roundup significantly decreased the proboscis extension response to water at 3 h after treatment at all tested concentrations (p = 0.027, 1/2 × the recommended concentration; p = 0.018, 1 × the recommended concentration; p = 0.032, 2 × the recommended concentration). The highest percentage of proboscis extension response was 28.33% in the control groups, and the lowest was 6.67% in the Roundup treatment of 2 × the recommended concentration (Fig. 1).

### Roundup affected sucrose responsiveness

**Figure 2:**
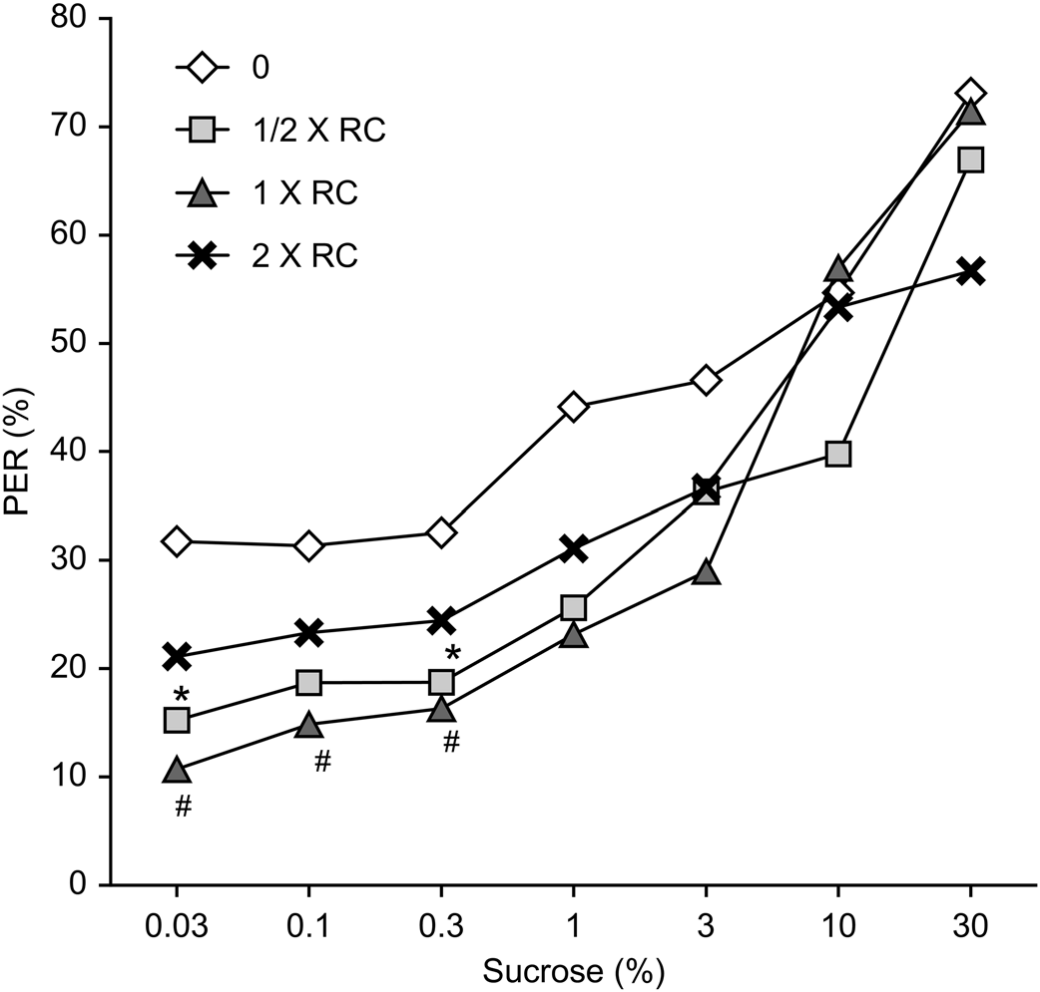
PER percentage responses of honeybees to increasing concentrations of sucrose. The values represent the means ± SEMs. df = 11. Significant diferences from the control are indicated by * for 1/2 × RC, # for 1 × RC (p < 0.05).

The proboscis extension response percentage increased with increasing sucrose concentration in the control and treatment groups. A significant decrease in the proboscis extension response percentage was detected in response to the 0.03% and 0.3% sucrose solutions after treatment with Roundup at 1/2 × the recommended concentration for 11 days (p = 0.028 and p = 0.015, respectively). In honeybees treated with Roundup at 1 × the recommended concentration, the proboscis extension response percentage in response to the 0.03%, 0.1% and 0.3% sucrose solutions was significantly lower than that of the control groups (p = 0.009, p = 0.047 and p = 0.007, respectively). Moreover, there was no significant effect of the proboscis extension response percentage for the ≥ 1% sucrose solution between the control and treatment groups. The groups of honeybees treated with 2 × the recommended concentration of Roundup did not exhibit any significant effects on the proboscis extension response percentage in any of the treatments (Fig. 2).

### Roundup affected olfactory learning and memory ability

**Figure 3:**
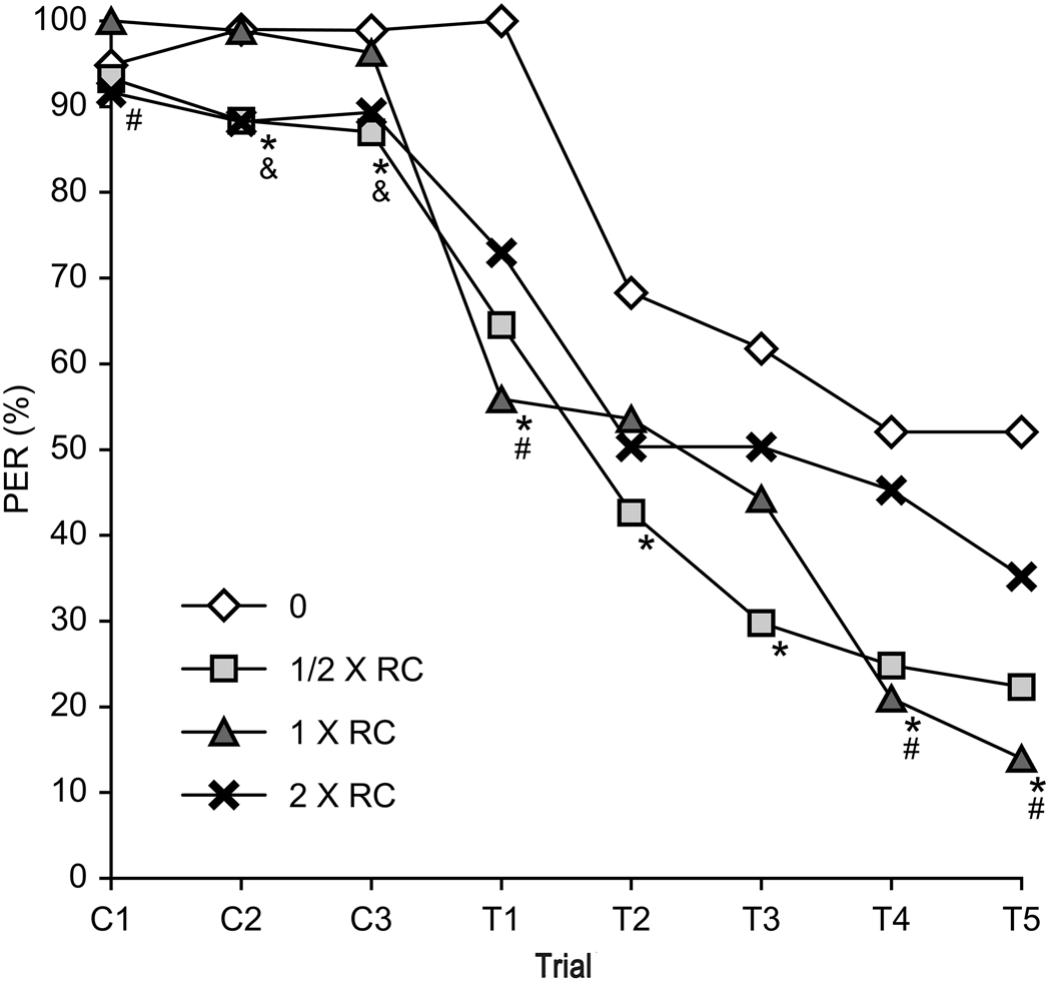
Olfactory learning and memory performance of honeybees after chronic exposure to Roundup. The values represent the means ± SEMs. df = 11. The positive responses at T1 of the control groups were scaled to 100%. Significant differences from the control are indicated by * for 1/2 × RC, # for 1 × RC, & for 2 × RC (p < 0.05).

The olfactory learning and memory ability of honeybees was expressed as the proboscis extension response percentage. Fixed honeybees were evaluated in the training phases C1, C2, and C3 and then in the test phases T1, T2, T3, T4 and T5. The proboscis extension response percentage of honeybees treated with Roundup at 1/2 × the recommended concentration indicated that the learning ability (the relationship between odour and sucrose feedback) during the second and third presentation of odour (C2 and C3) was significantly lower than that in the control groups (C2, p = 0.029; C3, p = 0.002). And compared with that in the control groups, the proboscis extension response percentage in the treatment group significantly decreased during all test phases (T1, p = 0.014; T2, p = 0.028; T3, p = 0.01; T4, p = 0.016; T5, p = 0.013). Compared with those in the control groups, the honeybees treated with Roundup at 1 × the recommended concentration presented a significantly increased proboscis extension response percentage at C1 (C1, p = 0.027) but a significantly decreased proboscis extension response at T1, T4 and T5 (T1, p = 0.001; T4, p = 0.041; T5, p = 0.011). A significant reduction in olfactory learning performance was also noted during C2 and C3 in honeybees treated with the highest dose of Roundup (2 × the recommended concentration) (C2, p = 0.011; C3, p = 0.013). The T1–T5 tests revealed no significant effect of the highest concentration of Roundup (Fig. 3).

### Roundup significantly reduced climbing ability

**Figure 4:**
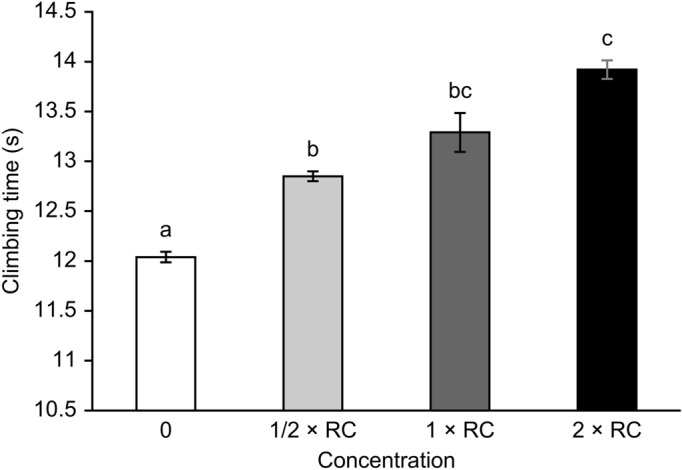
Time (s) spent by honeybees climbing along a 50-cm track after Roundup exposure. The values represent the means ± SEMs. df = 19. Signifcant differences from the control are indicated by letters of a, b, and c (p < 0.05).

The climbing ability of the honeybees was significantly affected by Roundup. Honeybees in the control groups required the shortest time to climb 50 cm, whereas those in the Roundup treatment groups at 2 × the recommended concentration required the longest time. During the climbing activity tests, honeybees treated with Roundup at all tested concentrations took longer to walk through the 50-cm track than did those in the control groups after 11 days of exposure (1/2 × the recommended concentration, p = 0.029; 1 × the recommended concentration, p = 0.031; 2 × recommended concentration, p = 0.008) (Fig. 4).

## Discussion

Roundup is one of the most widely used herbicides worldwide, and its global annual sales have always been the greatest. The LD_50_ of Roundup to honeybees was 309 µg/bee for 48 h in this study. This is similar to that in the material safety data sheet of Monsanto Company for Roundup Original Herbicide, which indicates the ingestion LD_50_ of *A. mellifera* for 48 h is 326 μg/bee^33^.

The doses of 7 and 14 μg/bee, which were equal to the 1/2 × , and 1 × recommended concentration respectively in this study, were inferior to 1/20 of the LD_50_ (309 µg/bee for 48 h). Therefore, both of the doses tested in this study were assumed to belong to a sublethal domain^34^.

Bees exposed to Roundup exhibited no difference in water responsiveness at 1 h in this study, which was similar to the response to other pesticides. The effects of acetamide and thiamethoxylamine on water responsiveness were not significantly different after 1 h of intragastric administration^31^. However, we found that the percentage of proboscis extension response decreased significantly after 3 h. It seemed that the formulation influenced the thirst of honeybees when the test time was prolonged. Water is very important for proper larval growth and development^35^. Brooding food consists of clear ingredients from the hypopharyngeal gland, possibly mixed with honey, digestive enzymes and water^36^. Therefore, the temperature of the hive and the feeding of the larvae may be affected if the honeybees are unwilling to collect water when affected by Roundup. Thompson et al. reported no significant effects of glyphosate on breeding, development and mean weight after treatment with pure technical-grade isopropylamine salt^20^.

Herbicide formulations always contain mixtures of inert ingredients with adjuvants/surfactants to enable effective penetration into target tissues^37–40^. It is well known that the commercially formulated glyphosate solutions and their inert ingredients, such as polyethoxylated tallow amine (POEA) in Roundup, are more toxic than glyphosate alone^40–44^. Thus, it posed higher risks to human health, especially among heavily-exposed applicators. It indicated that the toxicity of POEA within the commercially formulated glyphosate on honeybees need further study^45^. In addition, more independent and repeated experiments could make the results more accurate.

We found that Roundup at 1/2 × and 1 × the recommended concentration had negative effects on sucrose responsiveness. The effect was also previously observed by Herbert et al. Newly emerged honeybees exposed to 2.5 or 5 mg/L glyphosate for 15 days had lower sucrose responsiveness than did control honeybees^17^. Interestingly, the effect of Roundup exposure at 2 × the recommended concentration had no significant effect on sucrose responsiveness in this study. It seemed that Roundup at 2 × the RC balanced the effects of decreasing and increasing sucrose sensitivity, which also needs further study.

It is necessary to assess the learning and memory ability of worker honeybees involved in foraging. Tests are based on the ability to associate odours with rewards and remember this association after treatment with contaminated food. Compared with that of honeybees in the control groups, the learning ability of honeybees treated with Roundup was significantly affected at 1/2 × and 1 × the recommended concentration in this study. The negative effects of honeybees on olfactory associative learning have also been observed previously. Long-term exposure of sublethal concentrations of glyphosate (2.5 mg/L and 5 mg/L) hinders the dynamics of the ability to establish links between odour and reward^17^. Forager honeybees exposed to acute glyphosate doses showed impaired cognitive ability to retrieve and integrate information for successful foraging^17,32^.

The memory of honeybees in this study was significantly impaired after exposure to Roundup for 11 days at 1/2 × and 1 × the recommended concentration, which was different from the results of short-term memory in the report by Herbert et al. Those authors reported that long-term exposure to sublethal glyphosate had no effect on the establishment of short-term (15-min) memory^17^. It was speculated that memory was not affected because of the relatively low trace concentration and toxicity when pure GLY was used, as in the study by Herbert et al. Unexpectedly, although the learning ability was significantly inhibited in response to 2 × the recommended concentration, memory was not affected. These results are consistent with those of Gonalons et al. The effect of glyphosate combined with imidacloprid was weaker than that of imidacloprid alone. It seems that the action of imidacloprid is not detectable in the presence of glyphosate^46^. Foraging activities may require decision making based on information previously acquired through learning and memory^47,48^. Therefore, the results of our study suggest that chronic honeybee exposure to Roundup at 1/2 × and 1 × the recommended concentration may have a negative impact on the search and collection of resources and the coordination of foraging activities. The neurotoxin of Roundup also found in rainbow trout and rats. It evoked electro-olfactogram (EOGs) which indicated the formulation may had acted as an odorant, and generated a behavioral response in rainbow trout. This was avoided at glyphosate isopropyl amine concentrations ≥ 10 mg/L^27^. Tierney et al. (2006) also found significant electro-olfactogram reductions occurred within 10 min of exposure to 1 mg a.i./L and more rapidly with higher concentrations with the water-soluble herbicide glyphosate^28^. The effects that the prolonged immobility time and decreased time of climbing after Roundup exposure (3600 mg a.i./L, quivalent to 70 mg a.i./Kg/day) on rats were associated with oxidative stress and depressive-like behavior in offspring^29^.

We found that the climbing ability of honeybees significantly decreased after treatment with the recommended concentration of Roundup. The greater the concentration was, the faster the climbing ability decreased. Many of the tasks of honeybees involve climbing ability, so Roundup may affect the working ability of bees. Herbert et al. reported no changes in locomotive or directional activity between honeybees exposed to glyphosate at a concentration of 2.5 mg/L and those exposed to glyphosate at 5 mg/L for 15 days. The use of trace concentrations and pure glyphosate was less toxic than commercially formulated glyphosate without the surfactant. The impact of Roundup on honeybee locomotion was consist in other insects and soil invertebrates. Roundup residues (14.24 g a.i. /L, fresh or one-day old) did not alter the speed of locomotion of Pardosa spiders, but significantly reduced the crawled speed of Poecilus beetles exposed to residues than the control group^24^. Damselfly larvae exposed to Roundup (0.59 mg a.i./L and 1.19 mg a.i./L) had a higher foraging activity and a lower escape swimming speed, while it was only true at the highest concentration for glyphosate-exposed larvae^25^. In soil invertebrates, Roundup inhibited locomotion on *C. elegans* in a dose-dependent manner up to 86% at 10 µM (1.69 mg/L)^26^. Moreover, honeybees fed with glyphosate (0.5 µg/bee) solution were shown to spend more time flying home than were honeybees fed a pure sucrose solution or low concentrations of glyphosate (0.125 µg/bee and 0.25 µg/bee)^32^. Therefore, studying the effects of Roundup on honeybee navigation ability in response to recommended concentrations is necessary.

Honeybee behaviours might be severely affected when beekeepers fail to receive early warning and the bees in the colonies continue to forage the nectar and pollen sprayed with herbicides such as Roundup. And this herbicide could also alter the structures of royal jelly producing glands which can trigger damage to the development and survival of honeybee colonies^49^. In practice, rural farmers often double the concentration of herbicides to address resistance^10^. Therefore, it is necessary to evaluate the actual applications of commercially formulated glyphosate (CFG) at the recommended or higher concentration rather than the pure glyphosate on honeybees. In addition, China is a major beekeeping and bee product exporter worldwide, so it is strongly recommended that the relevant departments of the government establish an early warning and notification system to notify beekeepers before such herbicides are sprayed. Besides, commercially formulated glyphosate is widely used in agriculture and ecotoxins impact developing organisms differently than adults^25,29^. Increased amounts of attention should be paid to commercially formulated glyphosate because it not only is harmful to honeybees but also reportedly can cause human diseases after long-term use^40,50,51^.

## Conclusions

In this study, we provided new information on the influence of commercially formulated glyphosate at the recommended concentration on the behaviours of honeybees. Our findings showed that the water responsiveness, sucrose responsiveness, learning and memory ability and climbing ability of honeybees were affected by commercially formulated glyphosate at or below the recommended concentration. Additional research needs to be conducted to determine the effects of actual applications of commercially formulated glyphosate at the recommended or lower concentration rather than the pure glyphosate on honeybees.

## Materials and methods

### LD_50_

LD_50_ was tested to assess the dose level used in this study. Sampling sites were located at the Zhuifengshan Forest Apiary, Dachengzi town, Miyun district, Beijing. In order to avoid taking too many emerging bees at one time and affecting bee colonies, each experiment randomly selected six capped combs in six colonies from forty colonies and maintained in an incubator (these combs were put back into the original colonies after collection). Each cage (15 × 15 × 10 cm, with mesh on two sides) contained 70 honeybees that were captured within 24 h. A total of 4 cages were randomly selected for four treatments. The sucrose solution contained 41% glyphosate isopropylamine salt (Monsanto Roundup Original; 356 g of glyphosate acid equivalent per liter) at concentrations of 0, 0.72, 3.6, 7.2 g(s) of glyphosate acid equivalent per liter respectively (abbreviated as g/L). Plastic feeder were inserted vertically into the cages and changed daily. On the basis of the number of honeybees that survived daily, each honeybee was fed with an average of 33 µl of sucrose solution which can be guaranteed to be completely eaten to ensure that the average dose of each bee is fed, and then fed with sucrose solution (50%, w/w) and water ad libitum^34^. The honeybees were fed in an incubator whose temperature was 30 ± 1 °C and whose relative humidity was 65% ~ 70%. The insects in the control groups were fed 50% (w/w) sucrose solution and water. The total mortality rate was calculated at 48 h after treatment. Then six more combs from the rest colonies with capped pupae were randomly selected, and the entire experiment was repeated for 6 times in total and thirty-six colonies involved.

### Water responsiveness

The sampling sites and methods of honeybee collecting and feeding were the same as those above. Six combs with capped pupae were randomly taken from six healthy colonies and put them in the same incubator to take the emerging honeybees the next day. A total of 16 cages (70 bees/cage), 4 of which were randomly selected for water responsiveness assay (four treatment: 0, 1/2, 1, and 2 × recommended concentrations), and the rest for sucrose responsiveness, learning and memory and climbing assay (4 cages for four treatments in each behavioral experiment) respectively. Six combs were randomly selected again after such an experiment completed, and the entire experiment was repeated for a total of 3 times and 18 colonies involved (5 times for climbing assay).

At the beginning of the experiment, to keep the honeybees at the same age and adapt to the laboratory feeding, the honeybees were fed 50% sucrose for one week (during which each caged honeybee was fed with sufficient amounts of pollen, which was replaced daily). The recommended concentration of Monsanto Roundup Original was 50 ml–500 ml formulation solution in 30–40 L water according to different application^20,29,52,53^. The formulation in our study was applied at a rate equivalent to 50 ml formulation solution in 40 L water, which was the lowest recommended concentration and equal to 445 mg a.i./L (1 × recommended concentration). Beginning when they were 8 days old, the honeybees were continuously fed three concentrations of glyphosate (the 1/2 × , 1 × and 2 × recommended concentrations used in this study), which were equal to doses of 7, 14 and 28 μg/bee/day in 33 µl of 50% sucrose solution/day, on average. A honeybee age of 18 days old is the age at which nest working and foraging occur. Thus, 18 days old is a suitable age for testing a variety of working abilities and undertakings that are conducive to detecting the influence of the formulation^34^. The control groups were fed with a 50% sucrose solution. All tested honeybees were starved for 4 h before the test.

The honeybees were fixed in a tube such that the antennae and head could move freely. Water was placed on the antennae of the honeybees to detect the proboscis extension response (PER) as described in the study of El Hassani et al^31^. The honeybees were tested twice: at both 1 h and 3 h after treatment. Thirty honeybees were tested per replicate, and three replicates were used per treatment (0, 1/2 × , 1 × and 2 × recommended concentrations).

### Sucrose responsiveness

The bee rearing, treating and fixing methods are the same as previously mentioned methods. Water was first put on the antennae of the honeybees to determine whether the insects sucked water, eliminating the proboscis extension response phenomenon due to thirst. The proboscis extension response was then used to test honeybee sucrose responsiveness to increasing concentrations of sucrose solution (0.3%, 1%, 3%, 10%, 30%; w/v). The sucrose solution was absorbed and placed on the antennae of the honeybees to detect the proboscis extension response as described by El Hassani et al^31^. For each concentration, the percentage of proboscis extension response released by the honeybees was recorded.

### Olfactory learning and memory ability

The bee rearing, treating and fixing methods are the same as those mentioned above. Water was first placed on the antennae of the honeybees as described above. A 30% sucrose solution was used for the proboscis extension response assay, and the percentage of proboscis extension response was recorded. Linalool (Sigma, 95% purity) was used as the conditioned stimulus and was presented for 6 s first. During odour presentation, the proboscis extension response was elicited after 3 s by contacting the antennae with the sucrose solution (30%), and the same solution was immediately given as a reward. Conditioning trials were conducted three times (C1, C2, C3) at 20-min intervals. Five test trials (T1, T2, T3, T4, T5) were then conducted with linalool stimulation for 6 s without sucrose feedback as described previously^54^. Proboscis extension responses were also recorded at 20-min intervals.

### Climbing ability

Bee rearing and treating were the same as those mentioned above. After 11 days of continuous feeding, the honeybees were starved for 1 h before testing. A box (65 cm in length, 35 cm in width and 4 cm in height) was divided into 10 lanes (50 cm × 3.5 cm × 4 cm) with a fluorescent lamp on the top and covered with glass as described by Zaluski et al^30^. The tests were conducted in the dark and with the box tilted at 45°. One honeybee per lane was placed in the box, and the lamp was on. The time it took for a honeybee to climb 50 cm was recorded. Ten honeybees were tested in each trial (which was performed in five replicates) in the control and treatment groups.

### Statistical analysis

All the data were processed by SPSS 19 (IBM Corp., Armonk, NY, USA). The ingestion LD_50_ was determined on the basis of the mortality of honeybees for each dose via Probit analysis. The effects on water responsiveness, sucrose responsiveness, learning and memory and climbing were analyzed by ANOVA for both the Roundup-treated and control groups. And LSD (homogeneity of variance) or Tamhane’s T2 test (heterogeneity of variance) was performed. A difference was considered significant when the p-value was < 0.05.
